# Peroxiredoxin 4, A Novel Circulating Biomarker for Oxidative Stress and the Risk of Incident Cardiovascular Disease and All-Cause Mortality

**DOI:** 10.1161/JAHA.112.002956

**Published:** 2012-10-25

**Authors:** Ali Abbasi, Eva Corpeleijn, Douwe Postmus, Ron T. Gansevoort, Paul E. de Jong, Rijk O. B. Gans, Joachim Struck, Janin Schulte, Hans L. Hillege, Pim van der Harst, Linda M. Peelen, Joline W. J. Beulens, Ronald P. Stolk, Gerjan Navis, Stephan J. L. Bakker

**Affiliations:** Department of Epidemiology, University of Groningen, University Medical Center Groningen, Groningen, the Netherlands (A.A., E.C., D.P., H.L.H., R.P.S.); Department of Internal Medicine, University of Groningen, University Medical Center Groningen, Groningen, the Netherlands (A.A., R.T.G., P.E.d.J., R.O.B.G., G.N., S.J.L.B.); Department of Cardiology, University of Groningen, University Medical Center Groningen, Groningen, the Netherlands (P.v.d.H.); Julius Center for Health Sciences and Primary Care, University Medical Center Utrecht, Utrecht, the Netherlands (A.A., L.M.P., J.W.J.B.); Department of Research, Thermo Fisher Scientific/BRAHMS GmbH, Hennigsdorf, Germany (J. Struck, J. Schulte)

**Keywords:** cardiovascular disease, epidemiology, mortality, oxidative stress, peroxiredoxin 4

## Abstract

**Background:**

Oxidative stress has been suggested to play a key role in the development of cardiovascular disease (CVD). The aim of our study was to investigate the associations of serum peroxiredoxin 4 (Prx4), a hydrogen peroxide–degrading peroxidase, with incident CVD and all-cause mortality. We subsequently examined the incremental value of Prx4 for the risk prediction of CVD compared with the Framingham risk score (FRS).

**Methods and Results:**

We performed Cox regression analyses in 8141 participants without history of CVD (aged 28 to 75 years; women 52.6%) from the Prevention of Renal and Vascular End-stage Disease (PREVEND) study in Groningen, The Netherlands. Serum Prx4 was measured by an immunoluminometric assay in baseline samples. Main outcomes were: (1) incident CVD events or CVD mortality and (2) all-cause mortality during a median follow-up of 10.5 years. In total, 708 participants (7.8%) developed CVD events or CVD mortality, and 517 participants (6.3%) died. Baseline serum Prx4 levels were significantly higher in participants with incident CVD events or CVD mortality and in those who died than in participants who remained free of outcomes (both *P*<0.001). In multivariable models with adjustment for Framingham risk factors, hazard ratios were 1.16 (95% CI 1.06 to 1.27, *P*<0.001) for incident CVD events or CVD mortality and 1.17 (95% CI 1.06 to 1.29, *P*=0.003) for all-cause mortality per doubling of Prx4 levels. After the addition of Prx4 to the FRS, the net reclassification improvement was 2.7% (*P*=0.01) using 10-year risk categories of CVD.

**Conclusions:**

Elevated serum Prx4 levels are associated with a significantly higher risk of incident CVD events or CVD mortality and all-cause mortality after adjustment for clinical risk factors. The addition of Prx4 to the FRS marginally improved risk prediction of future CVD.

## Introduction

Experimental and clinical studies suggest that oxidative stress plays a key role in the pathogenesis of cardiovascular disease (CVD).^1–4^ Oxidative stress status is usually defined as overproduction of reactive oxygen/nitrogen species in imbalance with endogenous antioxidant defenses, which in turn result in increased oxidative damage.^5^ Several biomarkers, including target oxidation products and antioxidants, have been proposed for assessment of the level of oxidative stress, but clinical data examining association between markers and CVD independent of common risk factors are limited.^5–7^

Recently, peroxiredoxin 4 (Prx4), which is a secretable and stable isoform of the Prx family of antioxidant peroxidases,^8^ has been found in the circulation of humans. Prx4 can be precisely measured by a validated immunoassay.^9^ Previous evidence has shown abundant cellular antioxidant activity of Prx4 and other Prx isoforms in all mammals protecting against oxidative stress.^10–13^ So far, a limited number of small-scale studies have evaluated the association of the serum Prx4 with clinical data.^9,14^ In these studies, serum level of Prx4 was increased in septic patients compared with that of healthy individuals and was positively associated with well-established inflammatory markers like procalcitonin, C-reactive protein (CRP), and interleukin 6 (IL-6).^9,14^ Recently, a study in patients presenting to emergency departments showed the incremental prognostic value of Prxr4 to predict 30-day survival beyond usual risk predictors.^15^

We aimed to investigate whether serum Prx4 is a predictor of CVD and all-cause mortality. For this study, we used data of a large-scale observational cohort of the general population and examined the association of Prx4 with incident CVD events or CVD mortality, and all-cause mortality. Because a major clinical application of a biomarker lies within risk stratification and guided preventive strategies,^16–19^ we also evaluated the incremental predictive value of Prx4 above the Framingham risk score for the 10-year risk of CVD.

## Methods

### Study Population and Design

The study population was obtained from the Prevention of Renal and Vascular End-stage Disease (PREVEND) study, a Dutch cohort drawn from the general population (age range, 28 to 75 years) of the city of Groningen, the Netherlands, between 1997 and 1998. We have reported details of the study design and recruitment of participants elsewhere.^20,21^ Briefly, 40 856 individuals (47.8%) completed a questionnaire on demographics, history of cardiovascular and metabolic outcomes, medication use and pregnancy before their first visit, and collecting an early-morning urine sample in a vial to measure urinary albumin concentration. Those who were unable or unwilling to participate, individuals using insulin, and pregnant women were excluded. The baseline PREVEND participants were recruited from a total of 6000 individuals with a urinary albumin concentration ≥10 mg/L and a random control sample of individuals with a urinary albumin concentration <10 mg/L (n=2592).

In the baseline cohort, serum Prx4 assay was missing for 370 participants, leaving 8222 for the baseline cross-sectional analyses. The PREVEND study was approved by the local medical ethics committee at the University Medical Center Groningen and conformed to the principles outlined in the Declaration of Helsinki. All participants gave written informed consent.

### Clinical and Biomarker Measurements

In the baseline screening, study participants underwent 2 outpatient visits to assess demographics, anthropometric measurements, cardiovascular and metabolic risk factors, health behaviors, and medical family history and to collect two 24-hour urine samples on 2 consecutive days. Furthermore, information on medication use was substantiated with use of pharmacy-based data from all community pharmacies in the city of Groningen.^22^ Smoking and alcohol use were based on self-reports.

Hypertension was defined on the basis of self-report of diagnosis by a physician, measured hypertension (≥140/90 mm Hg systolic/diastolic blood pressure), or the use of blood pressure–lowering agents. Prevalent cases of type 2 diabetes were ascertained if 1 or more of the following criteria were met: (1) a fasting plasma glucose ≥7.0 mmol/L (126 mg/dL) or a random sample plasma glucose of 11.1 mmol/L (200 mg/dL), or (2) self-report of diagnosis by a physician, or (3) use of glucose-lowering agents according to a central pharmacy registration.^23^ Prevalent CVD was defined on the basis of self-reported physician diagnosis of cardiac, cerebral, and peripheral events by a physician. Kidney disease was defined on the basis of a history of kidney disease requiring dialysis or estimated glomerular filtration rate (eGFR) <60 mL/min per 1.73 m^2^. We used the Chronic Kidney Disease Epidemiology Collaboration (CKD-EPI) equation to calculate eGFR.^24^

In all participants, blood sample measurements for biomarkers were taken after an overnight fast. Serum Prx4 level was measured retrospectively in analogously stored baseline serum samples by a novel immunoluminometric assay, which was described previously.^9^ The functional assay sensitivity (interassay coefficient of variation <20%) was 0.51 U/L. The intraassay coefficient of variation was <8% throughout the range of Prx4 levels.^9,14^ Insulin was measured with an AxSym autoanalyzer (Abbott Diagnostics, Amstelveen, the Netherlands). Details on assays for total cholesterol, high-desnity lipoprotein (HDL) cholesterol, triglycerides, hs-CRP, and procalcitonin have been described previously.^25^ These baseline assays were performed in EDTA–plasma aliquots that had been stored frozen at −80°C without previous thawing and refreezing. Twenty-four-hour urinary albumin excretion (UAE)—given as the mean of the two 24-hour urine excretions—was measured by nephelometry with a threshold of 2.3 mg/L and intra- and interassay coefficients of variation <2.2% and <2.6%, respectively (Dade Behring Diagnostic, Marburg, Germany). All technicians were blinded to the participants' characteristics.

### Definition of Outcomes

In prospective data, we ascertained the main outcomes as follows: (1) incident CVD events, (2) incident CVD events or CVD mortality, and (3) all-cause mortality (up to January 1, 2009). Information (on hospitalization) for incident CVD events was obtained from PRISMANT, the Dutch national registry of hospital discharge diagnoses. The validity of this database has been shown to be good, with 84% of primary diagnoses and 87% of secondary diagnoses matching the diagnoses recorded in patients' charts.^26^ Data were coded according to the *International Classification of Diseases* (ICD), 9th revision, and the classification of interventions. The incident CVD events were classified as acute myocardial infarction (ICD code 410), acute and subacute ischemic heart disease (ICD 411), and occlusion or stenosis of the precerebral (ICD 433) or cerebral arteries (ICD 434), and the procedures including coronary artery bypass grafting or percutaneous transluminal coronary angioplasty and other vascular interventions, namely, percutaneous transluminal angioplasty or bypass grafting of aorta or peripheral vessels.

Data on mortality were obtained through the municipal registration. Cause of mortality was ascertained by linking the number of the mortality certificate to the primary cause of mortality as coded by the Dutch Central Bureau of Statistics. Survival time was defined as the period from baseline to the date of first incident CVD event, CVD mortality, date of death, or January 1, 2009. In the case when a person had moved to an unknown destination, the date on which the person was removed from the municipal registry was used as the censor date.^27^

### Statistical Analyses

Data are shown as mean±standard deviation (SD) or median (quartiles 1 and 3 [Q1, Q3]) for continuous variables, which were compared by 1-way analysis of variance or the Kruskal–Wallis test as appropriate. Frequency was used for categorical variables, which were compared by χ^2^ test across tertiles of Prx4. We calculated Spearman correlation coefficients of Prx4 with age, systolic blood pressure, body mass index (BMI), waist circumference, glucose, total cholesterol, HDL cholesterol, triglycerides, hs-CRP, procalcitonin, and 24-hour UAE. We used backward-elimination regression models to examine which of the clinical variables were independently associated with Prx4 as a dependent variable. The distribution of Prx4 was highly skewed. To normalize the distribution, we performed logarithmic transformation of the values of Prx4 before analyses. We used the logarithm base 2 (log_2_) to allow for interpretation of results per doubling of Prx4. Interpretation of results expressed per doubling of Prx4 seems more meaningful than interpretation per factor 10 change or per factor e change, which would have been the case if transformation according to base 10 or transformation according to the natural logarithm, respectively, would have been applied. We used Cox proportional hazards regression in crude and multivariable-adjusted models to examine the associations of Prx4 with incident CVD and all-cause mortality. We adjusted for age and sex in model 1. In model 2, we adjusted for Framingham risk factors including age, sex, smoking, systolic blood pressure, use of antihypertensive therapy, diabetes at baseline, total cholesterol, and HDL cholesterol.^28^ We tested the assumptions of proportional hazards for Cox regression models by Shoenfeld's global tests. Finally, in stepwise adjustments, we included alcohol use, triglycerides, high-sensitivity CRP (hs-CRP), and 24-hour UAE in model 2.

To assess incremental value of Prx4 for the risk prediction of CVD, we examined improvement of the prediction of CVD compared with the Framingham risk score. To do so, we calculated 10-year general CVD risk on the basis of the Framingham Risk Score^28^ and on the basis of a model with the Framingham Risk Score and log_2_ Prx4. Subsequently the models were compared in terms of the following measures, taking into account the time-to-event nature of the data^18,19,29,30^: (1) Harrell's C-statistic for the Cox proportional hazards regression to quantify the discrimination performance of the models (ability to distinguish between individuals with and without outcome); (2) net reclassification improvement (NRI) to examine if individuals with and without outcome were correctly reclassified (using the threshold values <6%, 6% to 20%, and ≥20% for categories of low-, medium-, and high risk, respectively^28,31^); and (3) integrated discrimination improvement (IDI), a continuous measure of reclassification.

Of the baseline sample of 8592 participants, 451 had a history of CVD. To do the prospective analyses, we first excluded these prevalent cases of CVD, leaving 8141 participants. For most baseline variables, fewer than 1% were missing; however, this was up to 8% for self-reported variables. We performed a single imputation with predictive mean matching for missing data. This method can be used for skewed data with <10% missingness, because it produces less biased estimates for a nonlinear model and imputations are in the metric of the observed data.^32,33^

Moreover, a weighted method was performed to compensate the baseline enrichment for the PREVEND participants with UAC ≥10 mg/L. Given the frequency of individuals with UAC ≥10 mg/L (24.4%) in our general population,^20,21,27^ we calculated the weight by sampling fractions. Those with UAC ≥10 mg/L had a weight=0.35, and those with UAC <10 mg/L had a weight=2.51.

Subsequently, we performed secondary analyses to take into account residual confounding. To do this, we incorporated other covariates that might be confounding of the association between Prx4 and the risk of incident CVD in combination with the Framingham risk factors (model 2). First, we further adjusted for BMI or waist circumference in separate models. Second, we adjusted for family history of CVD and examined the effects of kidney disease on this association. Third, we calculated metabolic syndrome, which was defined according to the National Cholesterol Education Program's Adult Treatment Panel III report criteria.^25^ And then we adjusted for metabolic syndrome or insulin in combination with variables in model 2. Next, we examined the association of Prx4 with each component of CVD events or CVD mortality including myocardial infarction, cerebrovascular disease, and cardiovascular mortality. In addition, we performed another analysis that included those who had a history of CVD. In total population, we further adjusted history of CVD in combination with the variables in model 2. And then we performed a similar analysis in participants with a history of CVD. We used Cox proportional hazards regression with fractional polynomials to search for the best-fitting functional form of Prx4 in the model for incident CVD (model 2).

Given the number of each event, we had 80% power at a 0.05 significance level to detect a hazard ratio=1.29 for myocardial infarction, 1.25 for cerebrovascular disease, and 1.23 for CVD mortality. All statistical analyses were carried out using IBM SPSS 19.0 and R version 2.10.1 (Vienna, Austria; http://75cran.r-project.org/).

## Results

### Baseline Characteristics

Baseline clinical characteristics of the total population and corresponding tertiles of serum Prx4 are summarized in Table 1. Median (Q1, Q3) Prx4 levels were 0.71 (0.45 to 1.16) U/L in men and 0.66 (0.42 to 1.08) U/L in women (*P*<0.001). Across tertiles of Prx4, those who had higher Prx4 levels were older, more obese, less frequent alcohol drinkers, and more likely to have a history of CVD, hypertension, and prevalent diabetes.

**Table 1.** Baseline Characteristics of Study Participants for Total Population's Corresponding Tertiles of Serum Peroxiredoxin 4*

Characteristic	Total		Tertiles†	
n	8222	2730	2671	2821

Peroxiredoxin 4, U/L	0.69 (0.44 to 1.12)	0.37 (0.37 to 0.44)	0.68 (0.59 to 0.78)	1.38 (1.10 to 1.97)

Age, year	49.2±12.7	47.1±11.9	48.7±12.4	51.8±13.2

Male sex, n (%)	4107 (50.0)	1276 (46.7)	1325 (49.6)	1506 (53.4)

History of CVD, n (%)	431 (5.4)	80 (3.0)	116 (4.5)	235 (8.7)

Family history of CVD, n (%)	3817 (50.2)	1292 (50.7)	1249 (50.3)	1276 (49.7)

Current smoker, n (%)	2787 (34.0)	1071 (39.4)	897 (33.7)	819 (29.1)

Ex-smoker, n (%)	2984 (36.4)	886 (32.6)	954 (35.9)	1144 (40.7)

Alcohol drinker, n (%)	6110 (74.7)	2121 (78.0)	2021 (75.9)	1968 (70.2)

BMI, kg/m^2^	26.1±4.2	25.3±3.8	26.1±4.2	26.8±4.5

Waist circumference, cm	88.5±13.0	86.0±12.1	88.3±12.8	91.2±13.8

Systolic blood pressure, mm Hg	124.5±19.6	121.0±17.5	124.0±19.3	128.3±21.1

Diastolic blood pressure, mm Hg	71.8±9.7	70.3±9.0	71.8±9.8	73.3±10.2

Antihypertensive therapy, n (%)	1282 (15.6)	294 (10.8)	396 (14.8)	592 (21.0)

Prevalent diabetes, n (%)	315 (4.0)	49 (1.9)	96 (3.7)	170 (6.2)

Fasting glucose, mg/dL	4.9±1.2	4.7±0.9	4.9±1.2	5.0±1.4

Total cholesterol, mmol/L	5.64±1.13	5.63±1.09	5.60±1.12	5.70±1.16

HDL cholesterol, mmol/L	1.32±0.40	1.37±0.40	1.32±0.39	1.27±0.40

Triglyceride, mmol/L	1.16 (0.85 to 1.68)	1.11 (0.81 to 1.56)	1.13 (0.83 to 0.164)	1.26 (0.90 to 1.88)

Metabolic syndrome, n (%)	1378 (18.2)	314 (12.3)	444 (17.9)	629 (24.5)

Fasting insulin, mmol/L	8.0 (5.6 to 12.1)	7.2 (5.2 to 10.2)	8.0 (5.5 to 11.9)	9.1 (6.2 to 14.3)

hs-CRP, mg/L	1.27 (0.55 to 2.96)	0.93 (0.42 to 2.08)	1.22 (0.55 to 2.82)	1.85 (0.79 to 4.27)

Procalcitonin, ng/mL	0.016 (0.013 to 0.20)	0.015 (0.013 to 0.018)	0.016 (0.013 to 0.019)	0.017 (0.014 to 0.021)

UAE, mg/24 h	9.45 (6.33 to 17.8)	8.76 (6.16 to 14.36)	9.11 (6.21 to 16.23)	10.87 (6.64 to 24.99)

CVD indicates cardiovascular disease; BMI, body mass index, which is the weight in kilograms divided by the square of the height in meters; HDL, high-density lipoprotein; hs-CRP, high-sensitivity C-reactive protein; and UAE, urine albumin excretion. Metabolic syndrome was defined according to the National Cholesterol Education Program's Adult Treatment Panel III report criteria.

* Data are mean (±SD) and median (quartiles 1 and 3) for continuous variables and percentage for categorical variables in complete baseline data set. For clinical variables, up to 1.2% was missing. For self-reported data, 0.4% to 7.8% was missing. For biomarkers, 0.2% to 6.4% was missing.

† *P*<0.001 for the comparison among all peroxiredoxin 4 tertile groups, except for total cholesterol (*P*=0.005) and family history of CVD (*P*=0.77).

Prx4 levels were positively correlated with age, BMI, waist circumference, systolic blood pressure, glucose, triglycerides, insulin, 24-hour UAE, and inflammatory biomarkers of hs-CRP and procalcitonin and were inversely correlated with HDL cholesterol (*P*<0.001 for all correlations) (Table 2). In the backward-elimination regression model, log_2_ Prx4 was positively associated with age (β=0.006, *P*<0.001), history of CVD (β=0.203, *P*=0.001), triglycerides (β=0.048, *P*<0.001), log_2_ hs-CRP (β=0.102, *P*<0.001), log_2_ UAE (β=0.050, *P*<0.001), and log_2_ insulin (β=0.102, *P*<0.001) and was inversely associated with female sex (β=−0.059, *P*=0.03), alcohol use (β=−0.083, *P*<0.001), and total cholesterol (β=−0.060, *P*<0.001) (Table 3).

**Table 2.** Spearman Correlation Coefficients of Serum Peroxiredoxin 4 With Baseline Variables*

Variable	Correlation Coefficient (95% CI)
Age	0.154 (0.131 to 0.177)

Systolic blood pressure	0.148 (0.117 to 0.164)

Body mass index	0.150 (0.128 to 0.170)

Waist circumference	0.167 (0.146 to 0.187)

Glucose	0.114 (0.088 to 0.135)

Total cholesterol	0.020 (0.000 to 0.044)

HDL cholesterol	−0.118 (−0.141 to −0.098)

Triglycerides	0.105 (0.080 to 0.133)

Insulin	0.181 (0.160 to 0.201)

hs-CRP	0.229 (0.205 to 0.249)

Procalcitonin	0.129 (0.107 to 0.153)

UAE	0.126 (0.104 to 0.150)

HDL indicates high-density lipoprotein; hs-CRP, high-sensitivity C-reactive protein; and UAE, urine albumin excretion.

* Data were available for 7638 to 8222 participants. We used the bootstrapping method to calculate 95% confidence intervals (CIs). *P* values were <0.001 for all correlations, except for total cholesterol (*P*=0.096).

**Table 3.** Association of Baseline Variables With Serum Peroxiredoxin 4 as Dependent Variable*

	Unadjusted		Age- and Sex Adjusted		Multivariable Adjusted†	
			
	β Coefficients (SE)	*P*	β Coefficients (SE)	*P*	β Coefficients (SE)	*P*
Age, per increase of 1 year	0.011 (0.001)	<0.001	0.011 (0.001)	<0.001	0.006 (0.001)	<0.001

Sex, female vs male	−0.081 (0.022)	<0.001	−0.061 (0.022)	0.005	−0.059 (0.027)	0.03

History of CVD, yes vs no	0.406 (0.049)	<0.001	0.261 (0.050)	<0.001	0.203 (0.052)	0.001

Smoking, yes vs no	0.032 (0.013)	0.02	0.006 (0.013)	0.67	—	—

Alcohol use, yes vs no	−0.172 (0.025)	<0.001	−0.158 (0.025)	<0.001	−0.083 (0.026)	0.001

BMI, per increase of 1 kg/m^2^	0.029 (0.003)	<0.001	0.021 (0.003)	<0.001	—	—

Waist circumference, per increase of 1 cm	0.010 (0.001)	<0.001	0.008 (0.001)	<0.001	−0.002 (0.001)	0.071

Systolic blood pressure, per increase of 1 mm Hg	0.007 (0.001)	<0.001	0.004 (0.001)	<0.001	0.001 (0.001)	0.074

Antihypertensive therapy, yes vs no	0.309 (0.030)	<0.001	0.194 (0.023)	<0.001	0.059 (0.035)	0.10

Prevalent diabetes, yes vs no	0.422 (0.057)	<0.001	0.291 (0.058)	<0.001	—	—

Total cholesterol, per increase of 1 mmol/L	0.027 (0.035)	0.006	−0.015 (0.010)	0.13	−0.060 (0.011)	<0.001

HDL cholesterol, per increase of 1 mmol/L	−0.227 (0.027)	<0.001	−0.204 (0.029)	<0.001	—	—

Triglycerides, per increase of 1 mmol/L	0.104 (0.011)	<0.001	0.081 (0.011)	<0.001	0.048 (0.013)	<0.001

Insulin, per increase of log_2_ unit	0.198 (0.013)	<0.001	0.171 (0.013)	<0.001	0.102 (0.017)	<0.001

hs-CRP, per increase of log_2_ unit	0.220 (0.023)	<0.001	0.114 (0.007)	<0.001	0.102 (0.008)	<0.001

Procalcitonin, per increase of log_2_ unit	0.127 (0.007)	<0.001	0.152 (0.024)	<0.001	0.049 (0.026)	0.10

24-hour UAE, per increase of log_2_ unit	0.101 (0.009)	<0.001	0.075 (0.009)	<0.001	0.050 (0.010)	<0.001

BMI indicates body mass index, which is the weight in kilograms divided by the square of the height in meters; SE, standard error; HDL, high-density lipoprotein; hs-CRP, high-sensitivity C-reactive protein; UAE, urine albumin excretion.

* Base-two logarithmically transformed serum level of peroxiredoxin 4 was the dependent variable.

† Backward selection was used when dropping nonsignificant variables (probability for removal was 0.10 by *F* test).

### Incident CVD and All-Cause Mortality

**Figure 1.**
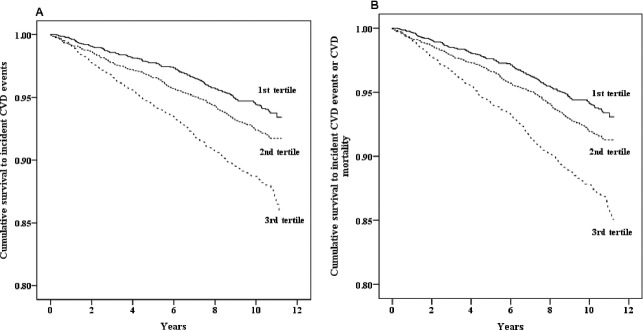
The cumulative probability of incident CVD events (A) and incident CVD events or CVD mortality (B) is shown by tertiles of Prx4. Data are shown for 8141 participants without CVD at baseline. CVD events were defined as a composite of nonfatal incident cardiac, cerebral, and peripheral vascular events. Overall, the log-rank tests were significant for all outcomes according to the tertiles of serum peroxiredoxin 4 (*P*<0.001). CVD indicates cardiovascular disease.

During median (Q1, Q3) follow-up of 10.5 (9.9 to 10.8) years, 663 participants (8.1%) developed incident CVD events, 708 participants (8.7%) developed incident CVD events or CVD mortality, and 517 participants (6.3%) died (of whom 135 died of cardiovascular causes). Median (Q1, Q3) Prx4 levels were 0.88 (0.54 to 1.45) U/L and 0.85 (0.53 to 1.46) U/L in participants who developed incident CVD events or CVD mortality and all-cause mortality, respectively. In Figure 1, the cumulative survival to incident CVD events and to incident CVD events or CVD mortality is shown based on tertile groups. The crude cumulative incidence rates (per 1000 person-years) and hazard ratios (HRs) with 95% confidence intervals (95% CIs) in crude and multivariable-adjusted models for the risk of developing incident CVD events, incident CVD events or CVD mortality, and all-cause mortality are shown in Table 3. Age- and sex-adjusted HRs (95% CIs) ranged from 1.32 (1.07 to 1.63) for incident CVD events to 1.40 (1.08 to 1.79) for all-cause mortality when comparing the highest tertile with the first tertile of Prx4 (*P* for trend <0.001). In a model adjusted for the Framingham risk factors, Prx4 was significantly associated with an increased risk of incident CVD and all-cause mortality. The proportional hazards assumptions were met for all models.

In further models, stepwise adjustment for alcohol use, triglycerides, hs-CRP, and 24-hour UAE minimally attenuated the associations of Prx4 with incident CVD events or CVD mortality. This was comparable to calculation of HR per doubling of Prx4 level for each outcome. We observed a 15% increased risk of incident CVD events independent of the Framingham risk factors per doubling of Prx4 level (HR 1.15, 95% CI 1.05 to 1.26). This was a 16% and a 17% increase for incident CVD events or CVD mortality (HR 1.16, 95% CI 1.06 to 1.27) and all-cause mortality (HR 1.17, 95% CI 1.06 to 1.29), respectively (Table 4). In subsequent analyses, the associations of Prx4 with the risk of either incident CVD or all-cause mortality were similar for men and women (data not shown).

**Table 4.** Association of Serum Peroxiredoxin 4 With Incident CVD Events, Incident CVD Events or CVD Mortality, and All-Cause Mortality (n=8141)*

	HR (95% CI) or No. of Cases (Incidence Rate) According to Tertiles of Prx4				
			
	1	2	3	HR (95% CI) Per Log_2_ Unit Increase*	*P*
Incident CVD events					

No. of cases, per 1000 person-years	151 (5.8)	204 (7.8)	308 (12.3)		

Unadjusted analysis	1.00	1.19 (0.95 to 1.48)	1.73 (1.40 to 2.13)	1.32 (1.21 to 1.44)	<0.001

Model 1	1.00	1.04 (0.84 to 1.30)	1.32 (1.07 to 1.63)	1.21 (1.10 to 1.33)	<0.001

Model 2	1.00	1.06 (0.84 to 1.32)	1.26 (1.01 to 1.57)	1.15 (1.05 to 1.26)	0.003

Model 2+alcohol use	1.00	1.06 (0.84 to 1.32)	1.25 (1.01 to 1.55)	1.14 (1.04 to 1.25)	0.004

Model 2+TG	1.00	1.06 (0.85 to 1.33)	1.28 (1.03 to 1.59)	1.16 (1.06 to 1.27)	0.001

Model 2+CRP	1.00	1.05 (0.84 to 1.31)	1.22 (0.98 to 1.52)	1.11 (1.01 to 1.22)	0.02

Model 2+UAE	1.00	1.05 (0.84 to 1.32)	1.25 (1.01 to 1.55)	1.15 (1.05 to 1.25)	0.003

Model 2+alcohol use, TG, CRP, and UAE	1.00	1.06 (0.84 to 1.32)	1.23 (1.00 to 1.53)	1.12 (1.02 to 1.23)	0.02

Incident CVD events or CVD mortality					

No. of cases, per 1000 person-years	160 (6.1)	215 (8.2)	333 (13.3)		

Unadjusted analysis	1.00	1.17 (0.94 to 1.45)	1.78 (1.46 to 2.19)	1.33 (1.23 to 1.45)	<0.001

Model 1	1.00	1.02 (0.82 to 1.27)	1.35 (1.10 to 1.66)	1.22 (1.12 to 1.33)	<0.001

Model 2	1.00	1.03 (0.83 to 1.29)	1.29 (1.05 to 1.59)	1.16 (1.06 to 1.27)	<0.001

Model 2+alcohol use	1.00	1.03 (0.83 to 1.29)	1.29 (1.04 to 1.58)	1.16 (1.06 to 1.26)	0.001

Model 2+TG	1.00	1.04 (0.84 to 1.30)	1.32 (1.07 to 1.62)	1.18 (1.08 to 1.28)	<0.001

Model 2+CRP	1.00	1.03 (0.82 to 1.28)	1.26 (1.02 to 1.56)	1.13 (1.03 to 1.23)	0.01

Model 2+UAE	1.00	1.03 (0.83 to 1.29)	1.29 (1.05 to 1.59)	1.16 (1.06 to 1.27)	0.001

Model 2+alcohol use, TG, CRP, and UAE	1.00	1.03 (0.83 to 1.29)	1.27 (1.03 to 1.57)	1.13 (1.03 to 1.24)	0.008

Incident all-cause mortality					

No. of cases, per 1000 person-years	117 (4.4)	160 (5.9)	240 (9.1)		

Unadjusted analysis	1.00	1.43 (1.10 to 1.86)	2.00 (1.56 to 2.57)	1.36 (1.23 to 1.50)	<0.001

Model 1	1.00	1.25 (0.96 to 1.62)	1.40 (1.08 to 1.79)	1.20 (1.08 to 1.33)	<0.001

Model 2	1.00	1.27 (0.98 to 1.66)	1.41 (1.09 to 1.82)	1.17 (1.06 to 1.29)	0.003

Model 2+alcohol use	1.00	1.27 (0.98 to 1.66)	1.41 (1.09 to 1.82)	1.17 (1.06 to 1.30)	0.002

Model 2+TG	1.00	1.26 (0.97 to 1.65)	1.39 (1.07 to 1.79)	1.16 (1.05 to 1.29)	0.004

Model 2+CRP	1.00	1.24 (0.95 to 1.62)	1.26 (0.97 to 1.64)	1.09 (0.98 to 1.21)	0.11

Model 2+UAE	1.00	1.27 (0.98 to 1.66)	1.40 (1.08 to 1.81)	1.15 (1.04 to 1.28)	0.006

Model 2+alcohol use, TG, CRP, and UAE	1.00	1.23 (0.94 to 1.60)	1.24 (0.96 to 1.61)	1.08 (0.97 to 1.20)	0.18

Cardiovascular disease (CVD) events were defined as a composite of incident cardiac, cerebral, and peripheral vascular events. Participants with a history of CVD were excluded. These associations did not differ by sex. Hazard ratios (HRs) with 95% confidence intervals (CIs) have been adjusted for age and sex in model 1 and for the Framingham risk factors including age, sex, smoking, systolic blood pressure, use of antihypertensive therapy, diabetes at baseline, total cholesterol, and HDL cholesterol in model 2. TG indicates triglyceride; CRP, C-reactive protein; and UAE, urine albumin excretion.

* Base-two logarithmically transformed Prx4, CRP, and 24-hour UAE were analyzed as continuous variables. Individuals with prevalent CVD were excluded.

To assess the incremental predictive value of Prx4 for the risk of CVD, we added Prx4 to the Framingham Risk Score as a continuous variable.^28^ In our data set, the Framingham risk score had a C-statistic (95% CI) of 0.80 (0.78 to 0.82) for the 10-year risk of CVD. The addition of Prx4 modestly improved the C-statistic to 0.81 (0.79 to 0.82, *P*=0.02) and led to IDI of 0.003 (*P*<0.001) and NRI of 2.7% (95% CI 0.7% to 4.7%; *P*=0.01) (Table 5). In patients without incident CVD events or CVD mortality, use of Prx4 reclassified 1% and 4% of participants in lower- and higher-risk categories (<6% and >20%), respectively. In patients with incident CVD events or CVD mortality, use of Prx4 reclassified 2% and 4% of participants in lower- and higher-risk categories, respectively.

**Table 5.** Reclassification of Participants for the 10-Year Risk Prediction of Cardiovascular Disease Corresponding to the Framingham Risk Score and After Adding Serum Peroxiredoxin 4*

	Framingham Risk Score With Prx4			
	
Framingham Risk Category	Low Risk	Intermediate Risk	High Risk	Reclassification (%)
In participants without outcome				

Low risk	4647	38	0	1.0

Intermediate risk	207	1984	81	13.0

High risk	0	23	494	4.0

In participants with outcome				

Low risk	104	2	0	2.0

Intermediate risk	9	299	24	10.0

High risk	0	9	213	4.0

Total sample				

Low risk	4751	40	0	1.0

Intermediate risk	216	2283	105	12.0

High risk	0	32	707	4.0

* Corresponding to the Framingham risk score and after adding serum peroxiredoxin 4, low risk denotes <6%, intermediate risk 6% to 20%, and high risk >20% for the 10-year of cardiovascular disease. Net reclassification index was 2.7 (95% CI 0.7 to 4.7; *P*=0.01), and integrated discrimination improvement was 0.0032 (95% CI 0.0014 to 0.0049; *P*<0.001).

### Secondary Analyses

Tables 6 and 7 show the results of secondary analyses. Separately, adjustment for BMI or waist circumference in combination with the Framingham risk factors (ie, model 2) did not materially change the association of Prx4 with risk of incident CVD events or CVD mortality. Moreover, our results adjusted for both kidney disease and family history of CVD were similar to those of model 2. In addition, further adjustment for metabolic syndrome or insulin in model 2 did not affect the association. We also investigated whether Prx4 was associated with the components of incident CVD events or CVD mortality including myocardial infarction, cerebrovascular disease, and CVD mortality. After adjustment for the variables in model 2, HR (95% CI) in the highest tertile compared with the first tertile of Prx4 was 1.03 (0.71 to 1.50), 1.28 (0.85 to 1.93), and 1.22 (0.71 to 2.12) for myocardial infarction, cerebrovascular disease, and CVD mortality, respectively.

**Table 6.** Association of Serum Peroxiredoxin 4 With Incident Cardiovascular Events or CVD Mortality (n=8141)

	HR (95% CI) by Tertiles of Peroxiredoxin 4 (U/L)		
	
Model	1	2	3
Adjusted for model 2	1.00	1.03 (0.83 to 1.29)	1.29 (1.05 to 1.59)

Adjusted for model 2+BMI	1.00	1.03 (0.83 to 1.28)	1.29 (1.05 to 1.59)

Adjusted for model 2+waist circumference	1.00	1.03 (0.83 to 1.28)	1.31 (1.06 to 1.59)

Adjusted for model 2+family history of CVD	1.00	1.04 (0.83 to 1.29)	1.30 (1.06 to 1.60)

Adjusted for sex, age, smoking+metabolic syndrome	1.00	1.03 (0.82 to 1.28)	1.38 (1.12 to 1.76)

Adjusted for model 2+insulin	1.00	1.04 (0.83 to 1.30)	1.31 (1.06 to 1.61)

Adjusted for model 2+kidney disease	1.00	1.03 (0.83 to 1.29)	1.30 (1.05 to 1.60)

Hazard ratios (HRs) with 95% confidence intervals (95% CIs) have been adjusted for model 2, in which the Framingham risk factors age, sex, smoking, systolic blood pressure, use of antihypertensive therapy, diabetes at baseline, total cholesterol, and HDL cholesterol were included. Kidney disease was defined on the basis of a history of kidney disease requiring dialysis or estimated glomerular filtration rate (eGFR) below 60 mL/min per 1.73 m^2^. We used the Chronic Kidney Disease Epidemiology Collaboration (CKD-EPI) equation to calculate eGFR. BMI indicates body mass index; CVD, cardiovascular disease; and HDL, high-density lipoprotein.

**Table 7.** Association of Serum Peroxiredoxin 4 With Incident Myocardial Infarction, Cerebrovascular Events, and Cardiovascular Mortality (n=8141)

	Tertiles of Peroxiredoxin 4, U/L		
	
	1	2	3
Incident myocardial infarction			

No. of cases, %	51 (5.8)	63 (7.8)	94 (10.1)

Unadjusted HR (95% CI)	1.00	1.04 (0.71 to 1.52)	1.43 (1.00 to 2.06)

Multivariate-adjusted HR (95% CI)*	1.00	0.98 (0.67 to 1.43)	1.06 (0.74 to 1.53)

Incident cerebrovascular disease			

No. of cases, %	39 (6.1)	58 (8.2)	88 (13.3)

Unadjusted HR (95% CI)	1.00	1.00 (0.64 to 1.55)	1.73 (1.16 to 2.58)

Multivariate-adjusted HR (95% CI)*	1.00	0.93 (0.60 to 1.45)	1.28 (0.85 to 1.91)

Incident cardiovascular mortality			

No. of cases, %	28 (1.0)	44 (5.9)	63 (9.1)

Unadjusted HR (95% CI)	1.00	1.48 (0.84 to 2.59)	2.10 (1.23 to 3.58)

Multivariate-adjusted HR (95% CI)*	1.00	1.35 (0.77 to 2.36)	1.38 (0.80 to 2.36)

* Hazard ratios (HRs) with 95% confidence intervals (95% CIs) have been adjusted for model 2, in which the Framingham risk factors included age, sex, smoking, systolic blood pressure, use of antihypertensive therapy, diabetes at baseline, total cholesterol, and HDL cholesterol.

In another analysis, we examined the association of Prx4 with risk of incident CVD after adjustment for the variables in model 2 and a history of CVD in the total population. The adjusted HR (95% CI) in the highest tertile compared with the first tertile of Prx4 was 1.32 (1.11 to 1.59) for incident CVD events or CVD mortality. In participants with a history of CVD, the adjusted HR (95% CI) for the variables in model 2 in the highest tertile compared with the first tertile of Prx4 was 1.07 (0.73 to 1.56) for incident CVD events or CVD mortality (n=181).

**Figure 2.**
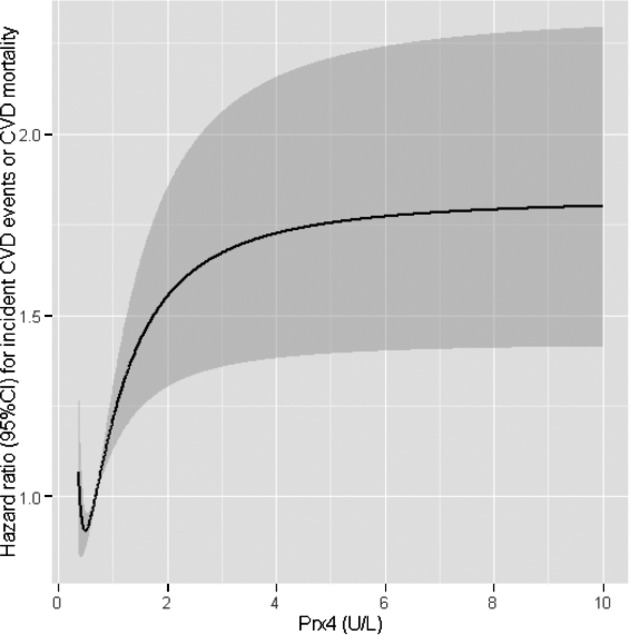
The relationship of peroxiredoxin 4 with incident CVD events or CVD mortality. Data are shown for 8141 participants without CVD at baseline. The plotted hazard ratio (95% CI) was adjusted for the Framingham risk factors and centered on the Prx4 median value. The optimal transformation of Prx4 was one in which the terms (Prx4)^1/2^ and (Prx4)^1/2^×ln(Prx4) were incorporated. CVD indicates cardiovascular disease.

Figure 2 depicts the relationship of continuous Prx4 with incident CVD events or CVD mortality. We plotted for Framingham risk factor–adjusted (model 2) HRs and their 95% CIs as a function of Prx4. The optimal transformation was one in which the terms (Prx4)^1/2^ and (Prx4)^1/2^×ln(Prx4) were incorporated. The solid line demonstrates that after a slight decrease in risk with levels slightly higher than the lowest ones that can be detected, the risk associated with increasing levels of Prx4 steeply increases until a plateau is reached with high levels of Prx4. Incremental value for risk prediction with the addition of (Prx4)^1/2^ and (Prx4)^1/2^×ln(Prx4) to the model rather than log_2_-linear transformed Prx4 (C-statistic=0.81, 95% CI 0.79-0.82; IDI=0.004, *P*<0.001; NRI=3.6%, 95% CI 1.5% to 5.7%, *P*<0.001) was slightly higher, but very similar to that with log_2_-linear transformed Prx4.

## Discussion

In this study, we demonstrated that serum Prx4, a circulating biomarker with antioxidant properties, was associated with the most common risk factors of CVD in a large general population cohort enriched with individuals with microalbuminuria. We found it to have an statistically significant positive association with age, history of CVD, systolic blood pressure, antihypertensive therapy, triglycerides, hs-CRP, UAE, and procalcitonin, wherea there was an inverse association with alcohol use and total cholesterol. Moreover, higher serum Prx4 levels were associated with significantly higher risk of incident CVD and all-cause mortality. For potential clinical application, we examined the incremental predictive value of Prx4 compared with the Framingham risk score. In particular, Prx4 modestly improved prediction for the 10-year CVD risk when added to the Framingham risk score in terms of discriminative ability and net reclassification.

Our findings showing the associations of serum Prx4 level with cardiovascular risk factors and events support previous clinical studies suggesting a role of oxidative stress in the pathogenesis of CVD.^1,2,4,34^ In an earlier study, a higher urinary excretion of oxidative stress indices was reported in individuals with renovascular hypertension, which was correlated with endothelium-dependent vasodilatation.^4^ Another study showed the independent association of oxidized low-density lipoprotein with the incidence of metabolic syndrome.^2^ In line with this, genotypes and serum activity of paraoxonase 1, an HDL-related antioxidant, has been shown to be associated with systemic oxidative stress and cardiovascular outcomes in humans.^1^ We now extend accumulating information obtained from animal and human studies on Prx4 of the family of thiol-dependent antioxidants in this era. An animal model of type 1 diabetes has indicated that Prx4 may have a pivotal role in the suppression of apoptosis and the proliferation of progenitor cells to protect against oxidative stress-induced β-cell dysfunction.^12^ In line with this, higher gene expression of Prx4 has been found in the islets of a high-fat diet model of β-cell dysfunction^35^ and downregulated expression of Prx4 in islets in diabetic mice with chronic hyperglycemia.^36^ Moreover, recent studies have suggested that Prx4 might be involved in the protection against celiac disease and cancer in the pancreas and lung with increased expression and production of Prx4 in the related human tissues.^12,37,38^ Consistently, Prx1, another member of the Prx family, has also shown to be involved in a broad range of oxidative stress-related outcomes in the cardiovascular system.^39,40^

Importantly, Prx4 is the only secretable member of the family in animals and humans.^8,11^ Recently, clinical data have shown that Prx4 levels were increased in sera of septic patients compared with healthy individuals.^14,15^ Consistent with this, we showed the relation between Prx4 and inflammatory markers and also explored its relation with measures of adiposity, blood pressure, glycemia index, lipids, and albuminuria. All these markers are underlying in central biological pathways of metabolic traits and CVD.^27,41^ Other recent data from patients who presented to emergency departments showed that serum Prx4 level was increased in nonsurvivors and that there was improved prognostic information for survival at 30 days when it was added to a clinical risk score.^15^ One main explanation for these findings may be that concomitant production and secretion of Prx4 can be augmented to protect against the increased rate of oxidative stress in relation to adverse cardiovascular and metabolic conditions.^42^ Interestingly, in secondary analyses with fractional polynomials, we found support for the notion that very low levels of Prx4 compared with somewhat higher levels are associated with a slightly increased risk. Possibly this makes up a subgroup of subjects with constitutively low levels of Prx4, which predisposes to CVD. The high risk with more elevated levels of Prx4 is consistent with a compensatory increase in Prx4 in response to existing oxidative stress. At the cellular level, Prx4 may promote antioxidant activity via several pathways, such as nuclear factor kB,^43^ p53,^44^ thromboxane A2 receptor,^45^ and NF-E2-related factor 2 (Nrf2).^46^ Despite equivocal findings of supplementing antioxidant vitamins to prevent cardiovascular and metabolic diseases,^1,47^ a recent trial^48^ showed an effective intervention of a specific antioxidant, that is, an Nrf2 antagonist that is related to Prx4 pathway, against decline of renal function in patients with chronic kidney disease and type 2 diabetes. Therefore, this might be an opening line for novel therapeutic targets and monitoring tools for metabolic traits and CVD.

Next, we assessed the clinical application of Prx4 for the risk prediction of CVD. To do this, we used information from a general 10-year risk of CVD based on the Framingham risk score^28^ and examined whether Prx4 might have incremental predictive value above this risk score. We did not develop new models, but chose an appropriate approach in prediction research by using the information retained in the existing prediction model.^19,30,31,49^ Of note, there are usually missing values for baseline data in an observational study. We had <10% of data missing, and we used a single imputation and predictive mean matching. Imputation for missing values will increase the power and precision of a study and minimize the risk of biased findings.^50^ Finally, we used reclassification measures, which are more sensitive and more clinically relevant than C-statistic alone.^18,19,49^ For CVD, the addition of Prx4 to the Framingham risk score marginally improved prediction in terms of discrimination and reclassification. Overall, the addition of Prx4 correctly reclassified 2.7% of participants for risk of CVD obtained by the Framingham risk score. Further studies are warranted to confirm our findings for this oxidative stress biomarker in different settings^17^ and among individuals with other comorbidities such as diabetes.

There are some limitations to this study that should be addressed. Our cohort predominantly comprised white adults, and it is therefore unknown whether our findings can be generalized to nonwhites. The PREVEND cohort was enriched for microalbuminuria at baseline. However, a weighted method was performed to compensate for this, which did not affect the results. Moreover, we investigated the association of only baseline and not serial circulating levels of Prx4 with the future risk of outcomes of interest. The extent of within-individual variation over time might affect the statistical power of a study.^51,52^ For example, higher variation can lead to less power, and then the estimated risk is attenuated using a single Prx4 measurement. Although we accounted for confounding of traditional cardiovascular risk factors, a potential for uncontrolled or residual confounding would be plausible. Finally, we had no data on other oxidative stress markers such as oxidized low-density lipoprotein and homocysteine to compare their predictive values with that of Prx4.

In conclusion, our results suggest that higher serum Prx4 levels are associated with most cardiovascular risk factors including albuminuria and hs-CRP in a general-population cohort study. Moreover, higher serum Prx4 levels were associated with a significantly higher risk of incident CVD events or CVD mortality and all-cause mortality after adjustment for traditional cardiovascular risk factors. Prx4 marginally improves the 10-year risk prediction for CVD when added to the Framingham risk score.
